# Examining Method Effect of Synonym and Antonym Test in Verbal Abilities Measure

**DOI:** 10.5964/ejop.v11i3.865

**Published:** 2015-08-20

**Authors:** Wahyu Widhiarso, Haryanta

**Affiliations:** a Universitas Gadjah Mada, Yogyakarta, Indonesia; Aalborg University, Aalborg, Denmark; Academy of Special Education, Warsaw, Poland

**Keywords:** method variance, confirmatory factor analysis, synonyms and antonyms test

## Abstract

Many researchers have assumed that different methods could be substituted to measure the same attributes in assessment. Various models have been developed to accommodate the amount of variance attributable to the methods but these models application in empirical research is rare. The present study applied one of those models to examine whether method effects were presents in synonym and antonym tests. Study participants were 3,469 applicants to graduate school. The instrument used was the Graduate Academic Potential Test (PAPS), which includes synonym and antonym questions to measure verbal abilities. Our analysis showed that measurement models that using correlated trait–correlated methods minus one, CT-C(M–1), that separated trait and method effect into distinct latent constructs yielded slightly better values for multiple goodness-of-fit indices than one factor model. However, either for the synonym or antonym items, the proportion of variance accounted for by the method is smaller than trait variance. The correlation between factor scores of both methods is high (r = 0.994). These findings confirm that synonym and antonym tests represent the same attribute so that both tests cannot be treated as two unique methods for measuring verbal ability.

Measurement process comprises a number of elements, including the attribute being measured, the instrument being used to measure, the methods of using the instrument, and the unit of measurement. In contrast to the physical sciences, which generally have established and consistent measurement methods, measurement in the social sciences is still evolving, and there is therefore no single exact method for measuring individual attributes (Urbina, 2004). Individual attributes such as, for example, intelligence, can be measured by using a number of different instruments and methods. To assess an individual's true psychological complexity, multiple instruments that employ a variety of methods for collecting data are used. For example, in assessing job performance, the primary instrument that measures employee performance may be supplemented by information from external sources such as peers and supervisors.

In the field of measurement, method has a wide meaning, comprising all ways of measuring (Kline, 2011). The method can refer to the source of information (e.g., self, others) (Urbina, 2004) the format scale (e.g., Likert, semantic differential) (Kenny, 1976), the direction of the statements in the scale (e.g., positive or negative) (Marsh, 1996), or the overall format of the instrument (Marsh, Asci, & Thomas, 2002). For this reason, different instruments that measure the same construct (e.g., self-esteem)—such as Rosenberg’s Self-Esteem Inventory and Coppersmith’s Self-Esteem Inventory—could be perceived as different methods for measuring self-esteem.

The existence of these various measurement methods inspired Campbell and Fiske (1959) to develop the multi-trait multi-method (MTMM) analysis of construct validity. An instrument is assumed to attain high construct validity if there is a strong correlation between two instruments that measure similar attributes using different methods, while different instruments that measure different attributes have low correlations. This type of testing assumes that the method of measurement should not affect the scoring of the attribute of interest: if the obtained correlation is high, then both instruments can be assumed to be valid. This approach implies that methods of measurement are interchangeable.

This concept of interchangeability of method was challenged by the discovery of the person specific method, meaning that individuals respond differently to different methods. For example, some individuals are likely to get lower scores on paper-and-pencil tests than on computer-based test, meaning that different methods may affect the individuals’ obtained score. The assumption that methods were completely interchangeable was also challenged by the discovery of method effects, meaning that using different methods may unintentionally require different skills. For example, a test of literacy may give very different results if administered in a paper-and-pencil form or on a computer.

Another crucial method effect was introduced by Marsh (1996), who found that instruments for measuring self-esteem gave very different results if the items were worded positively (e.g., “I am proud of myself”) or negatively (e.g., “I am not proud of myself”). Currently, in the scale development literature, different item stems with different intensities and emphases are used to minimize response style bias (e.g., acquiescence bias). For this reason, assessments of mood, for example, should not only include items that measure positive mood (e.g., “I feel happy”) but also negative mood (e.g., “I feel sad”). Both items are parallel and interchangeable—individuals who truly have a positive mood state will endorse the first item but not endorse the second—this mix of positively and negatively phrased items should reduce response bias.

However, this technique assumes that mood follows a bipolar continuum, which has not found to be strictly the case in bi-dimensional models, as happiness and sadness, for example, can occur simultaneously (Steyer & Riedl, 2004). Mood is not the only example of this measurement-related concern; in many psychological measurements, attributes that theoretically stand on the opposite ends of a continuum can occur simultaneously. Established examples of this include trust and distrust (Lewicki, McAllister, & Bies, 1998) as well as masculinity and femininity (Udry, 2000). Factor analysis conducted on such scales reveal the existence of item groups based on the type of item, as opposed to on the attribute of interest. These findings support Marsh’s (1996) finding that items presented positively or negatively do not only function as a different measurement methods, but may also assess different attributes. Clearly, different measurement methods may result in bias, which can sometimes only be detected after when the measurement instrument has been administered.

When testing the construct validity of a new instrument, researchers must distinguish between method effects (differences due to the use of different methods) and construct effects (differences due to actual differences in the attribute of interest). Method effects are often also referred to as method-related sources of variance, which help researchers to pinpoint what variance in the measure can be attributed to the methodology or other types of systematic measurement error (Podsakoff, MacKenzie, Lee, & Podsakoff, 2003). Method variance is operationalized as correlations among the uniqueness’s of variables measured by the same method (Conway, 2002) that represents form of systematic measurement error that contaminates the validity of measurements.

Under MTMM construct validity testing, method-related variance is seen as a residual that must be minimized; this form of validity testing attempts to maximize construct-related variance and minimize method-related variance. Method-related variance is seen as a residual that is unrelated to construct-related variance. This is similar to regression analysis, which also seeks to minimize residuals. The residuals in regression analysis are random, and not correlated with true score variance. This explains the effect of the predictor on the criterion. Following the principle that residuals must be random and do not correlate to any variable, method-related variance must not relate to any variable, including the construct measured. A low correlation between the construct and method variance implies that the construct is independent from the selected method of measure; in this case, the method used is just one alternative among many alternative methods that could be used to assess the construct, and this method could be replaced by others. In contrast, a high correlation between the construct and method variance means that the construct must be assessed using the same method that was already used, as changing the method will reduce the information provided about the construct being measured. Researchers seek to minimize the role of method effects due to conceptual and methodological constructs, so as to isolate the true underlying variance.

Recent literature suggests that using different methods can strongly impact the construct being measured (Pace, 2010). For example, researchers may use two methods, self-report and peer judgment, to measure social support. These methods are not parallel, and cannot replace each other, meaning that eliminating one of those methods will change the operational definition of social support. For example, removing the peer judgment in the preceding example will change the attribute being measures to perceived social support, as it is being assessed only through subjective self-report. This example illustrates how different methods sometimes cannot be eliminated or even separated when measuring psychological attributes. Researchers in this area therefore need an analytical procedure to assess the correlation between the constructs being measured and the methods being employed.

## Synonym and Antonym Test

Tests measuring cognitive abilities usually focus on the ability involved in thinking (i.e., reasoning, perception, memory, verbal ability, math, and problem solving). Such tests pose questions designed to assess test-takers’ potential ability to use mental processes to solve school- or work-related problems. In the work-related context, cognitive ability testing can determine the extent to which an individual’s performance on the job is related to their learning, problem-solving and decision-making capabilities (Allworth & Passmore, 2008).

Verbal abilities tests usually measure verbal knowledge and verbal reasoning. There are many types of tests available to measure verbal abilities (e.g., synonym, antonym, analogy, reading comprehension, and sentence completion). All sub-types are not used in every test measuring cognitive abilities; two well-known examples of this are the Scholastic Aptitude Test (SAT) and the Graduate Record Examination (GRE). The selection of the types of sub-tests depends on the theory, purpose, and test-taker characteristics chosen by test developers. For example, in 1994 major changes in both content and procedures were implemented in the SAT: antonym items were eliminated from the verbal section, reading comprehension items were made more complex, and sentence completion items were added (Zwick, 2007).

The present study focused on two verbal abilities tests: synonyms and antonyms. Although prior studies have assumed that synonym and antonym tests are substitutive methods that both measure verbal comprehension (e.g., Ward, 1982), we believe that these two sub-tests measure different domains. Two words are synonyms if they have similar meanings. In a synonym question, respondents must choose the word that is closest in meaning to the given word (e.g., happy–glad). Individuals are instructed to look for the best match among the choices given, not necessarily a perfect match. Antonyms are two words that have opposite meanings. In an antonym question, individuals must choose the word or phrase that most nearly means the opposite of the given word (e.g., happy–sad). Synonyms and antonyms have paradigmatic and syntagmatic relationships, respectively, between the two matched items. According to Rapp (2002), words with a paradigmatic relationship are normally the same part of speech, whereas words with a syntagmatic relation can be (but need not be) the same part of speech. Further, although the definitions of synonym and antonym are very similar, the strategy for handling antonym questions is different from that for answering synonym questions. Synonymy and antonymy are semantic relations of a very different logical nature, opposition of meaning is not simply the extreme case of difference in meaning (Lyons, 1968). The opposite of synonymity is heteronymy while the opposite of antonymy is lack of opposition (Hermann, Conti, Peters, Robbins, & Chaffin, 1979).

There are several considerations in assessing verbal abilities using word relationship questions: the key answer should be most similar in meaning or most opposite in meaning, excerpted text should not be used when one must analyze word relationships within answer choices, only grade-level appropriate words found within the text should be assessed, and the question should contain clear and sufficient context for determining the meaning of the assessed words (Florida Department of Education, 2010). The clarity of word context differs between synonym and antonym questions, with synonym questions providing a clearer verbal context and more specificity than antonyms (Freedle & Kostin, 1990). Antonyms questions is not merely described set phrases (e.g., long and short, thick and thin) but antonym pairs co-occur across a large range of different phrases (Paradis & Willners, 2011).

The synonym and antonym tests are unique in measuring verbal comprehension. This uniqueness is associated with the differences in the skills individuals need to be able to take these two tests. At a certain level, individual only requires knowledge about vocabulary to successfully take the synonym test; however, individuals need both knowledge of vocabulary and reasoning to succeed with the antonym test, as the antonym test assesses one’s knowledge about vocabulary specific to the context and meaning of the word. This notion is supported by several authors. Many tests employ synonym test purposed to measure breadth of vocabulary whereas antonyms measure analogical thinking (Arjona-Tseng, 1993). Solving antonym questions require broad domain of cognitive skills (Phillips, 2013), whereas solving synonym question require narrow domain of cognitive skills. A study conducted by Jeon and colleagues (2009) examined different brain activity when individual solving synonym and antonym questions. Solving synonym activated brain that associated with mental processes of searching and selecting target words that have similar features in certain dimensions. In other sides, solving antonym questions activated brain represent mental process of reversing the semantic meaning in one dimension and finding opposite features. As a consequence due to differences in measurement domain, items of both tests have different level of difficulty. Antonym items may be easier to be processed than synonym items (Sabourin, 1998).

Synonym and antonym test are non-substitutable methods, since they are both associated with different domain attribute of verbal abilities being measured. We therefore hypothesized that the synonym and antonym cannot be understood as interchangeable methods in measuring verbal ability, since both test measure different domain of verbal ability. To test our hypothesis, we employed several analyses: comparing model fit of CFA models that assuming synonym and antonym is the same versus different method and examining the correlation between true score of synonym and antonym test.

## Method

### Participants

Participants of the present study were 3,469 graduate school applicants (Age range: 24–46 years) for Universitas Gadjah Mada. Data were taken between 2010 to 2011 in six cities in Indonesia: Yogyakarta, Jakarta, Balikpapan, Makassar, Mataram, and Surabaya.

### Instruments

The instrument used in the present study was the Academic Potential Test for Graduate Students (PAPS) Form A1, developed by Azwar, Suhapti, Haryanta, and Widhiarso (2009). The PAPS aims to assess students’ potential to succeed in an academic setting, and consists of three components, with a total of 120 items: verbal, quantitative, and logical. The verbal component contains synonym, antonym, analogy, and reading comprehension items (Azwar et al., 2009); however, the present study looked only at the synonym and antonym components. Each subtest consists of 10 items, with five alternative answers for each item. For the synonym test, participants are instructed to choose the word that has most nearly the same meaning as the target word. For the antonym test, participants are instructed to choose the word that has the most nearly opposite meaning to the target word. For both of these tests, the total score is the total number of items that have been answered correctly. Among the five possible answers, only one answer is correct.

### Analysis Procedure

**Figure 1:**
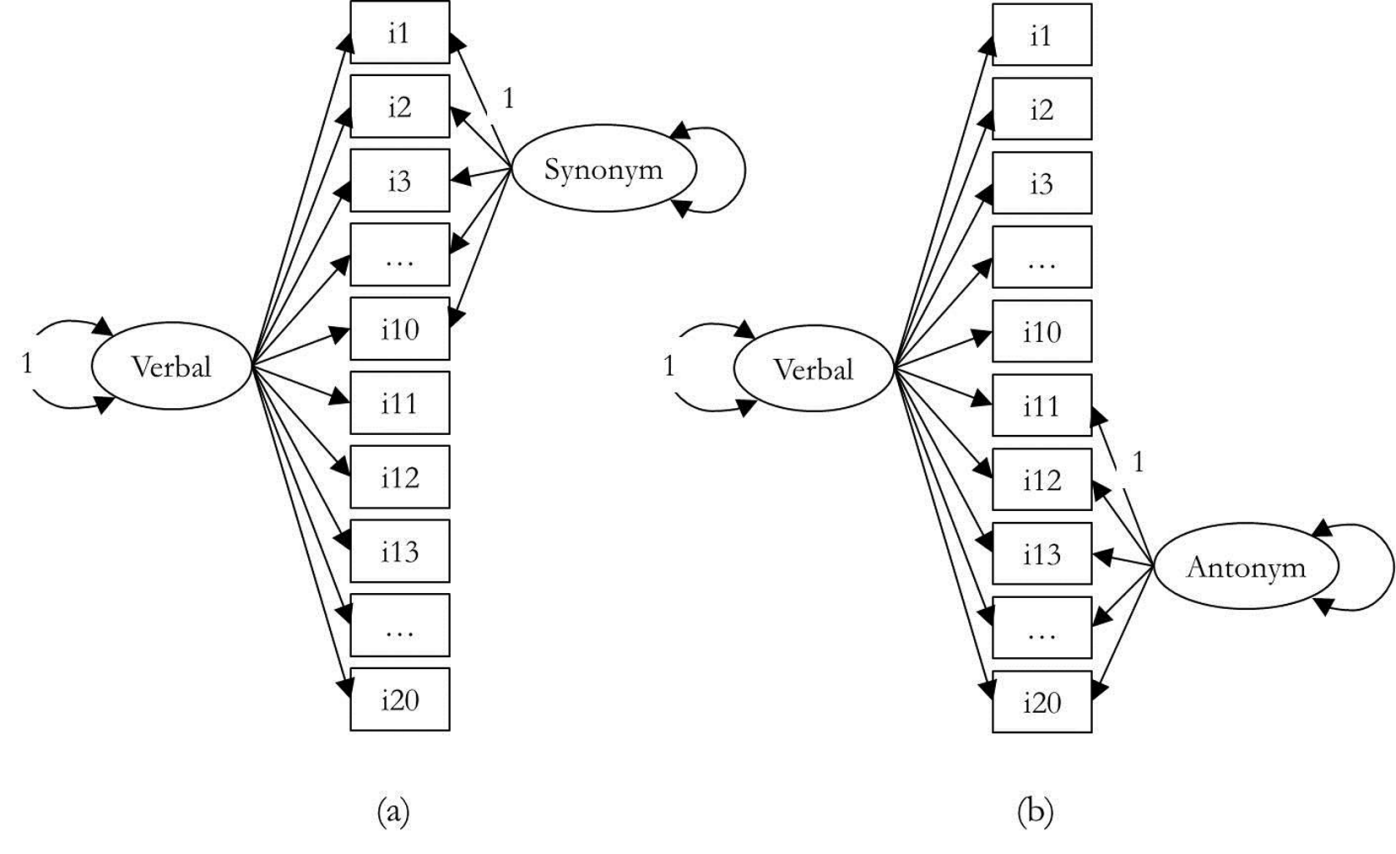
Path diagram of a CT-C(M–1) model for the Verbal Ability data. *Note*. Items 1-10 are synonym items, and items 11-20 are antonym. All loadings are freely estimated. A double-headed arrow on a single variable indicates its variance. For the sake of simplify, measurement error on each item is omitted. The model is identified by fixing the variance of the reference factor and the loading of one item on the non-reference factor to 1.0.

We hypothesized that these two tests represented two distinct methods and measured different domains of verbal abilities. Analyses were performed using CFA, using models derived from the four models presented in Figure 1. Three models were tested: Model 1 is a null model that involves a single factor simultaneously comprised of synonym and antonym items. Model 2 consist of two factors; the first factor consists of all synonym and antonym items (verbal abilities factor), while second factor consists of only items from the same method either synonym or antonym (method factor). In the Model 2(a), antonym items were selected as the reference method so that the model contains a non-reference (method) factor for the synonym items. In the Model 2(b), synonym items were selected as the reference method so that the model contains a non-reference (method) factor for the antonym items.

We estimated chi-square (χ2) statistics representing the Likelihood Ratio (LR) that compares the relative fit of saturated model and restricted model (model being tested). Smaller χ2 values indicate a slightly better fit than larger χ2 values. Since this test is sensitive to sample size, it is important to rely on a multiple-index fitting strategy (Jöreskog & Sörbom, 1988). Besides (χ2), there are many indices proposed by authors (see Hu & Bentler, 1999). This study used The Comparative Fit Index (CFI) and The Tucker Lewis Index (TLI) with ranging from 0 to 1. High value of these indexes indicating a great degree of variance in the data accounted for by the model. This study also used the Root Mean Square Error of Approximation (RMSEA), an index that represented represents the amount of variance not accounted for by the model. The RMSEA value below 0.06 suggests satisfactorily model fit.

## Results

### Model Testing and Comparison

MPLUS 6.12 (Muthén & Muthén, 2005) was used to analyze all models using weighted least squares means and variance adjusted (WLSMV). Because empty answers were treated as incorrect answers, then there is no missing value available in the data. The results indicated that all models yielded acceptable fit. The RMSEA values for all models were below .06, CFI and TLI values were both above .90 (Hu & Bentler, 1999). Since chi-square test is easily affected by sample size, the evaluation of model–data fit was based on these four goodness-of-ﬁt indices (RMSEA, CFI, and TLI). Analysis showed that the three tested models produced satisfactory fit-indices (see Table 1). The results from test comparison between the model using the chi-square test for difference testing in MPLUS (i.e., DIFFTEST) found model fit differences, the CT(CM-1) either use a synonym (∆χ^2^ = 44.389; *p* <. 01) or antonym (∆χ^2^ = 69.690; *p* < .01) items as a reference, produces a better fit value. Results from the model comparison showed that all alternative models (Model-2) provided slightly a better fit than the baseline model (Model 1).

**Table 1:** Statistics for Fit Indices of the Three Tested Confirmatory Analysis Models

Model		χ^2^	df	P	RMSEA	CFI	TLI	WRMR
Model-1	One-factor model	378.907	170		0.019 (0.016 - 0.021)	0.955	0.950	1.259
Model-2a	CT-C(M-1) model	335.597	160	p < 0.01	0.018 (0.015 - 0.020)	0.962	0.955	1.180
Model-2b	CT-C(M-1) model	301.466	160	p < 0.01	0.016 (0.013 - 0.019)	0.970	0.964	1.107

*Note*. RMSEA = root mean square error of approximation; CFI = comparative fit index; TLI = Tucker Lewis Index; WRMR = weighted root mean square residual.

### Factor Loading

A more precise assessment of trait and method-related variance can be established by examining individual parameter estimates (i.e., factor loading) (Byrne, 2009). Hence, this section will compare standardized factor loadings between trait and method factor. Standardized loadings can be interpreted as correlations between indicators and factors in the CC(M-1) model, because the reference factor is uncorrelated with all method factors pertaining to the same trait-method unit in this model (Geiser, Eid, West, Lischetzke, & Nussbeck, 2012). In the CTC(M-1) model, one method is chosen as standard (reference) and the specific factors are treated as method factors. The factor loadings obtained when the antonym items (Model 2a) and synonym items (Model 2b) are used as reference method are shown in Table 2. Result from Model 2a suggests that half of synonym items factor loadings are all smaller than the trait factor loadings. Result from Model 2b suggests that all antonym items factor loading are smaller than trait factor loadings. These imply that either for the synonym and antonym items, the proportion of variance accounted for by the method is smaller than the proportion of variance accounted for by the verbal ability.

**Table 2:** Factor Loadings for each Tested CFA Model

No.	Baseline		Model 2(a)				Model 2(b)			
	Verbal Ability		Verbal Ability		Method Factor		Verbal Ability		Method Factor	
I1	0.34	(0.027)	0.36	(0.03)	0.19	(0.06)	0.34	(0.03)		
I2	0.46	(0.026)	0.48	(0.03)	0.07*	(0.06)	0.47	(0.03)		
I3	0.37	(0.037)	0.38	(0.04)	0.10*	(0.06)	0.35	(0.04)		
I4	0.01*	(0.035)	0.02*	(0.04)	0.18*	(0.08)	-0.03*	(0.04)		
I5	0.06*	(0.027)	0.06*	(0.03)	0.34	(0.06)	0.05*	(0.03)		
I6	0.20	(0.025)	0.21	(0.03)	0.07*	(0.08)	0.19	(0.03)		
I7	0.16	(0.024)	0.17	(0.03)	0.09*	(0.07)	0.15	(0.03)		
I8	-0.43	(0.098)	-0.44	(0.10)	0.33	(0.06)	-0.45	(0.10)		
I9	0.14	(0.034)	0.14	(0.04)	0.32	(0.06)	0.13	(0.03)		
I10	0.27	(0.024)	0.29	(0.03)	0.25	(0.06)	0.26	(0.02)		
I11	0.23	(0.027)	0.18	(0.03)			0.23	(0.03)	0.04*	(0.05)
I12	0.32	(0.024)	0.32	(0.03)			0.32	(0.02)	-0.08*	(0.05)
I13	0.47	(0.024)	0.46	(0.03)			0.47	(0.02)	0.33	(0.06)
I14	0.91	(0.027)	0.89	(0.03)			0.91	(0.03)	0.47	(0.08)
I15	0.41	(0.022)	0.34	(0.03)			0.41	(0.02)	0.11*	(0.05)
I16	0.83	(0.026)	0.84	(0.03)			0.83	(0.03)	0.20	(0.05)
I17	0.70	(0.024)	0.70	(0.03)			0.70	(0.02)	0.13	(0.05)
I18	0.46	(0.023)	0.39	(0.03)			0.47	(0.02)	0.21*	(0.17)
I19	0.38	(0.024)	0.31	(0.03)			0.38	(0.02)	0.09*	(0.06)
I20	0.40	(0.021)	0.35	(0.03)			0.41	(0.02)	0.33	(0.06)

*Note*. Standard errors for estimated parameters are shown in parentheses.

*Factor loadings are not significantly different from zero (*p* < .05).

Since in the CTC(M-1) model, the high factor loadings of the non-reference indicators on the reference factor show the degree of convergent validity between methods used for measure, our results suggests that synonym and antonym test have achieved convergent validity. Results of analysis show that individual’s score of each test represents trait variance more than method variance.

### Correlation Between Tests

**Figure 2:**
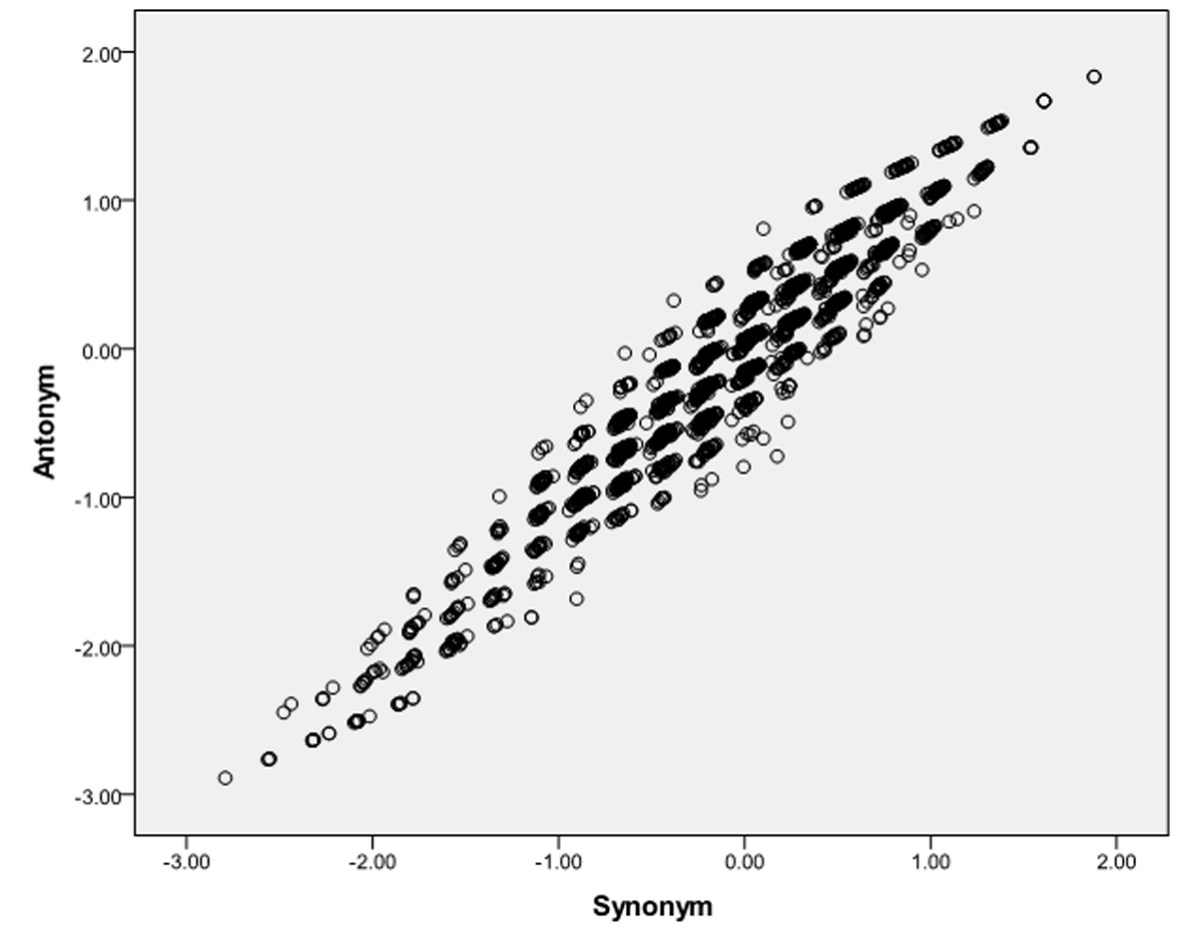
Scatter plot of factor scores from Synonym and Antonym Test.

In order to test the correlation between individual’s latent score of synonym and antonym test, we fitted model that consists of two factors (synonym and antonym) to the data. The tested model yields a good fit to the PAPS data: χ^2^ (41, *N* = 3.469) = 379.098; *p* < .05; RMSEA = 0.019 (0.016 - 0.021) CFI = 0.955; TLI = 0.949. The correlation between synonym and antonym factor is very high (*r* = 0.994). This high correlation suggests that between synonym and antonym items have little uniqueness in addition to their shared construct of verbal ability. The cross plotting the person scores from the two test is depicted in Figure 2. From this figure we can see that the points are scattered closely to a straight line and the correlation is high, thus it can be concluded that the two sets of items do indeed measure the same construct. Using this model, we found that synonym items were more difficult (*M* = 0.070) antonym (*M* = -0.496).

## Discussion

Synonyms and antonyms are two test or methods for measuring verbal abilities (verbal knowledge and reasoning). The aim of the present study was to examine whether these two test measure different abilities. If the synonym and antonym measure different attributes then they can be seen as two complementary methods for measuring verbal ability. If both measure the same attributes then they have the possibility to be seen as two methods that can be interchangeable. Our hypothesis was not supported, as the analysis confirmed that synonyms and antonyms are two the same methods that measure similar domains of cognitive abilities. Although, measurement models that separated synonym and antonyms as distinct latent constructs yielded slightly better goodness-of-fit indices than the null model, a comparison of factor loadings across traits and methods suggests that the proportion of trait variance exceeds method variance in the most of the items. The null model in this study was a one-dimensional model and the alternative models were CTC(M-1). Thus, although evidence of discriminant validity appeared to be fairly good at the matrix level since model fit comparison suggest CTC(M-1) model better than one factor model; a more in-depth examination at the individual parameter level reveals low method effect associated with synonym and antonym test.

Synonym tests follow the synonymy concept; two words are said to be synonyms if one can be used in a statement in place of the other without changing the meaning of the statement (Jarmasz & Szpakowicz, 2003). There are many strategies at how to choose the best answer, the most common being to interpret the distance between the nodes that correspond to the items being compared: the shorter the path, the more similar the items (Resnik, 1995). Our study findings were supported by the fact that synonym tests are more complicated than antonym tests, as finding an opposite meaning requires strong reasoning skills as well as synonym test. This was why separating the synonym and antonym tests produced the model with the best fit. Synonym items are particularly easy to answer because they are modeled by preference materials of all kinds, in which a main word is paired with the words similar to it. However, the relationships underlying antonym items are quite a bit more varied, and are not systematically compiled (Turney, Littman, Bigham, & Shnayder, 2003).

The strategies for handling antonym questions are quite different than those for handling synonym questions, an opposite meaning can be found in several ways. The usual way is to instantly locate the opposite meaning directly, based on colloquial language. However, this method is hampered by the presence of distractor items in the possible responses, which are deliberately chosen to catch test-takers using this strategy. This example illustrates how the procedures needed to answer antonym-type questions require different cognitive effort than synonym-type questions. Antonym question have different patterns than synonym questions. These patterns can be understood by people who have large vocabularies, good reasoning skills, and the ability to connect these two skills. Test-takers then have to think of synonyms of quick, such as fast, and then find the antonym of that secondary word among the list of possible responses. When individuals are asked to articulate the relationship between the words broad and road, they might consider a number of possibilities. Many possible relationships would need to be considered, depending on the context (Turney et al., 2003), unlike for synonym questions. Antonym items have limited verbal contexts (Freedle & Kostin, 1990), requiring individuals to interpret what context might be appropriate to connect the term in the question and possible answers.

Because this research found that variance associated with specific methods in synonym and antonym test do not exceed variance associated with attribute of interest (i.e., trait variance), we conclude that the unique variance proportion produced by the two tests is small. Although synonym and antonym tests were found to be mutually substituted, using these types of tests in a measurement instrument should be done only after considering the different skills and attributes measured by these two types of tests. While both tests can be used to measure cognitive abilities—synonym and antonym tests are among the best measures of verbal reasoning, they are complementary. Individuals learn most new words by inferring their meanings from the contexts in which the words are embedded, and then remembering and revising their understandings as they encounter the words repeatedly, in different contexts.
